# Antihypertensive Activity of* Leersia hexandra* Sw. (Poaceae) Aqueous Extract on Ethanol-Induced Hypertension in Wistar Rat

**DOI:** 10.1155/2019/2897867

**Published:** 2019-01-06

**Authors:** Danielle Claude Bilanda, Yannick Carlos Tcheutchoua, Paul Désiré Djomeni Dzeufiet, Daniel Lauré Dongmo Fokou, Yannick Bekono Fouda, Théophile Dimo, Pierre Kamtchouing

**Affiliations:** Department of Animal Biology and Physiology, Laboratory of Animal Physiology, University of Yaoundé I, P.O. Box 812, Yaoundé, Cameroon

## Abstract

*Leersia hexandra *(*L. hexandra*) is used in traditional medicine to treat many diseases including hypertension. This study aimed to evaluate the curative effects of the aqueous extract of* L. hexandra* on hypertension. Hypertension was induced in rats by oral administration of ethanol (5 g/kg/day) for five weeks. The animals were divided into 2 groups: one group of 5 rats receiving distilled water (10 mL/kg) and another group of 20 rats receiving ethanol. At the end of the 5 weeks of administration of ethanol, the animals were divided into 4 groups of 5 rats each: one group of hypertensive rats receiving distilled water (10 mL/kg), another one receiving nifedipine (10 mg/kg), and two groups of hypertensive rats receiving* L. hexandra* at doses of 100 and 200 mg/kg, respectively. The results showed that ethanol induced a significant increase in the mean arterial pressure (MAP) and heart rate of normotensive rats. The administration of the extract (100 and 200 mg/kg) or nifedipine caused a significant decrease of MAP compared to hypertensive rats. Ethanol induced a significant increase of lipid profile, the atherogenic index, creatinine, and transaminase activities. Ethanol also induced a significant decrease in serum HDL-cholesterol and antioxidant markers evaluated. Treatment of hypertensive rats with* L. hexandra* or nifedipine significantly improved lipid profile, hepatic and renal functions, and antioxidant status. The curative effect of* L. hexandra* extract on hypertension is probably related to its antihypertensive, hypolipidemic, and antioxidant activities, which justifies its empirical use in the treatment of hypertension.

## 1. Introduction

Improvement of the living and working conditions of man made possible by new technologies has led to unprecedented behavioral changes. A sedentary lifestyle, greater ease of doing daily chores, and consumption of high-calorie or oversalty foods, cigarettes, or alcohol are important contributors to the emergence of conditions such as obesity, diabetes, and high blood pressure (HBP). Hypertension (HT) is a cardiovascular disease whose complications (hypertrophic cardiomyopathy, hypertensive encephalopathy, malignant retinopathy, malignant nephroangiosclerosis, arteriosclerosis, etc.) are the leading causes of death worldwide [1]. The global prevalence of hypertension in the adult global population was estimated in 2014 at 30% [1]. In sub-Saharan Africa, the prevalence of hypertension was estimated at 16.2% or 74.7 million hypertensive patients in 2013 [2]. Epidemiological data suggest that this proportion is expected to reach 68% or 125.5 million people in 2025 [2]. Prevalence growth in developing countries is probably related to changes in nutritional habits, sedentary lifestyle, or alcohol use [3]. Alcohol consumption is related in a dose-dependent manner to an increase in systolic and diastolic blood pressure. This increase is mainly observed when drinking more than 20 g/day for both men and women [4]. The treatment of hypertension has several axes including drugs and improving lifestyle. An antihypertensive treatment possibly associated with dietary and lifestyle measures considerably reduces the arterial pressure and consequently reduces cardiovascular morbidity and mortality [5]. These drugs have several limitations, namely, side effects, cost, and inaccessibility for some locations in developing countries. The search for new drugs, especially from natural products and manly plants, is of great interest for the development of more efficient and better tolerated drugs.

*Leersia hexandra* used in the current study is a medicinal plant belonging to the family of Poaceae with a wide occurrence in tropical and subtropical countries and the hot areas of Africa [6]. In the traditional pharmacopeia of Senegal,* L. hexandra* is used for the treatment of hemoptysis (rejection by the mouth of blood coming from the breathing apparatus) among weakened patients undergoing frequent coughing fits [6]. This plant is also used in Indonesia for the treatment of muscular tiredness [7]. In Cameroun, it is used traditionally for the treatment of hypertension. This study was carried out to evaluate the curative effects of the aqueous extract of the leaves and stems of* Leersia hexandra* on a model of hypertension induced by ethanol in rats.

## 2. Material and Methods 

### 2.1. Preparation of Plant Extract

The external parts of* L. hexandra *were collected at Dschang in the west region of Cameroon in March 2015. The plant material was authenticated at the National Herbarium, Yaoundé, by comparison with the existing voucher specimen N° 6850/HNC (YA) deposited by Mister Ngansop Tchatchouang, Eric. The plant was dried at room temperature and reduced to a powder. The powder (500 g) was put in 5 L of hot water (100°C), allowed to cool down, and filtered following the traditional method. The solution obtained after filtration with Wattman N° 3 filter paper was lyophilized and gave 19 g of the aqueous extract (3.8% yield).

### 2.2. Qualitative Determination of Compounds Contents of* L. hexandra *Using HPLC-DAD-HRESI-MS

#### 2.2.1. Sample Preparation

Aqueous preparations extracts were separately dissolved in HPLC grade methanol at a concentration of 5 mg/ml and then filtrated through a syringe-filter-membrane. Aliquots of 5 *μ*L were injected into the LC-DAD/MS Dionex Ultimate 3000 HPLC (Germany), used for performing the analyses.

#### 2.2.2. HPLC-MS Conditions

High resolution mass spectra were obtained with an OTOF Spectrometer (Bruker, Germany) equipped with a HRESI source and a UV-vis absorbance detector. The spectrometer was operated in positive mode (mass range: 100-1500, with a scan rate of 1.00 Hz) with automatic gain control to provide high-accuracy mass measurements within 2 ppm deviation using Na formate as calibrant. Mass spectra were simultaneously acquired using electrospray ionization in the positive ionization mode. The following parameters were used for experiments: spray voltage of 4.5 kV and capillary temperature of 200°C. Nitrogen was used as sheath gas (10 l/min). The spectrometer was attached to an Ultimate 3000 (Thermo Fisher, USA) HPLC system consisting of an LC-pump; UV traces were measured at 215, 218, 254, 280, and 330 nm and UV spectra*―*a Diode Array Detector (DAD)*―*were recorded between 190 and 600 nm, and the system also consisted of an autosampler (injection volume 5 *μ*l) and a column oven (35.0°C). The separations were performed using a Synergi MAX-RP 100A (50 x 2 mm, 2.5 *μ* particle size) with a H_2_O (+0.1% HCOOH) (A)/acetonitrile (+0.1% HCOOH) (B) gradient (flow rate 500 *μ*L/min). Samples were analyzed using a gradient program as follows: 95% A isocratic for 1.5 min and linear gradient to 100% B over 6 min and after 100% B isocratic for 2 min, the system returned to its initial condition (90% A) within 1 min and was equilibrated for 1 min.

#### 2.2.3. Identification of Peaks

Identification of all constituents was performed by HPLC-DAD-MS analysis and by comparing the UV and MS spectra and MS/MS fragmentation of the peaks in the samples with those of the data reported in the literature of the SciFinder database.

### 2.3. Drugs and Chemicals

All the drugs and chemicals used in this study were purchased from Sigma Chemical Company (St. Louis, MO, USA).

### 2.4. Animals

Twenty-five male Wistar rats of 6-8 weeks of age, weighing between 150 and 160 g, were housed in plastic cages and maintained in the animal house of the Department of Animal Biology and Physiology, Faculty of Science, University of Yaoundé I, Cameroon. Animals were maintained under standard laboratory conditions with a natural luminosity cycle, with free access to normal laboratory rat food and tap water. Prior authorization for the use of laboratory animals in this study was obtained from the Cameroon National Ethical Committee (Reg. N° FWA-IRD 0001954).

### 2.5. Animals Grouping and Treatments

The animals were divided into 2 groups. The first group made of 5 rats received distilled water (10 mL/kg/day), and the second group of 20 rats received ethanol 40° (5 g/kg) for 5 weeks. At the end of the 5 weeks, the first group was maintained and the second group divided into 4 groups of 5 rats each: one group of hypertensive rats receiving concomitantly ethanol and distilled water (10 mL/kg) and 3 groups of hypertensive rats receiving concomitantly ethanol and* L. hexandra* extract (at the doses of 100 and 200 mg/kg) or nifedipine (10 mg/kg, p.o.), respectively. Throughout the experiment, body weight was evaluated.

### 2.6. Hemodynamic Parameters Recording

At the end of the respective treatment, the arterial blood pressure and heart rate of all rats were recorded [8]. Briefly, each rat was anesthetized using an intraperitoneal injection of urethane (1.5 g/kg). The trachea was exposed and cannulated to facilitate spontaneous breathing. The arterial blood pressure was measured from the carotid artery via an arterial cannula connected to a pressure transducer coupled with a hemodynamic recorder Biopac Student Lab. (MP35) and a computer.

### 2.7. Blood and Organs Collection

Immediately after hemodynamic parameters recording, blood samples were collected from the abdominal artery and centrifuged at 3000 rpm for 15 min. The plasma obtained was kept at - 20°C for biochemical analysis. Thereafter, the heart, kidney, liver, and thoracic aorta were collected, washed in saline, weighed, and kept for assessment of oxidative stress markers.

### 2.8. Biochemical Analysis

The Mc Even solution was used to homogenize the heart and aorta while a Tris–HCl (50 mM) buffer solution was used for the liver and kidney (20%, w/v). Each homogenate was centrifuged at 3000 rpm for 25 min and stored at -20°C. Tissue protein concentration was assayed according to Gornall et al. [9] using the Biuret reagent. Malondialdehyde (MDA) was determined using the procedure of Wilbur et al. [10]. Superoxide dismutase (SOD) was determined using the method described by Misra and Fridovich [11]. Catalase was determined according to Sinha [12], whereas reduced glutathione (GSH) was determined using the method described by Ellman [13]. The serum concentrations of total cholesterol (TC), high density lipoprotein cholesterol (HDL), low density lipoprotein cholesterol (LDL), triglycerides (TG), and creatinine levels were determined using commercial diagnostic kits (Fortress, UK). The atherogenic index was calculated following the formula used by Youmbissi et al. [14]. The activities of alanine aminotransferase (ALT) and aspartate aminotransferase (AST) were also determined spectrophotometrically using commercial diagnostic kits (SGM Italia). For microscopic evaluation, a part of the investigated organs was fixed in 10% formalin for 7 days and paraffin-embedded for microscopical examination in accordance with routine laboratory procedure. Paraffin sections of 4 *μ*m were prepared and stained with hematoxylin and eosin (H&E) for histological examination. Morphometric measurements of the thickness of arteries were performed using ImageJ 1.3. Six arteries per animal were quantified, giving a total of 36 vessels per group.

### 2.9. Statistical Analysis

Results were expressed as the mean ± SEM. The difference between the groups was compared using one-way analysis of variance (ANOVA) followed by Tukey's post hoc test. A value of* p* < 0.05 was considered statistically significant. All analyses were performed using GraphPad Prism software version 5.03.

## 3. Results

### 3.1. Qualitative Determination of Compounds Contents of* L. hexandra *Using HPLC-DAD-HRESI-MS

**Figure 1:**
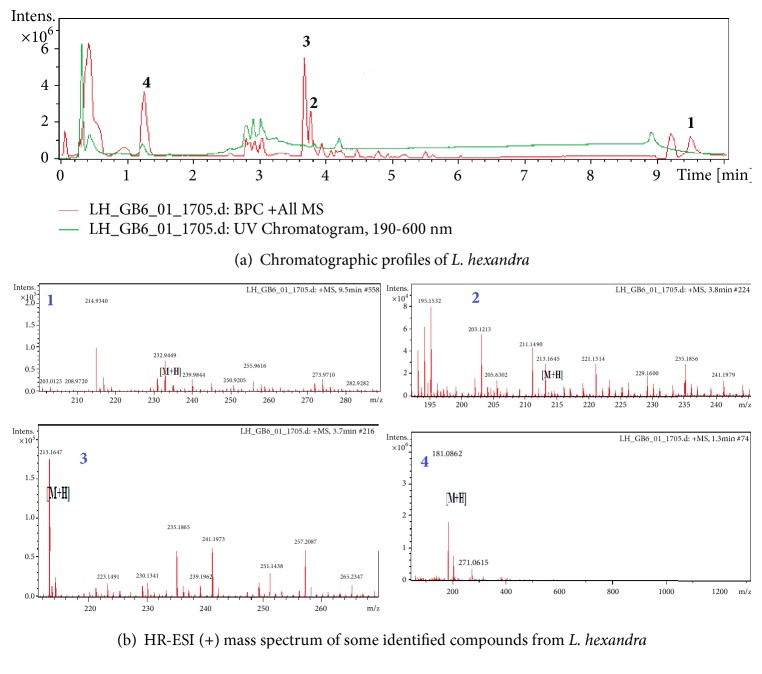


The extracted crude* L. hexandra* sample was analyzed by HPLC coupled to both diode array and mass spectrometry detectors. The latter was used with an electrospray ionization source in positive ion mode. A representative base peak chromatogram and all ions MS are shown in Figure 1 and Table 1 indicating that the used HPLC conditions allowed a good separation of a large percentage of compounds. The compounds were recognizable from their characteristic UV spectra, which were identified based on the HPLC-DAD-HRESI-MS data and subsequent confirmation by comparison with literature data.

### 3.2. Effects of the Aqueous Extract of* L. hexandra *on Body Weight Gain

**Figure 2:**
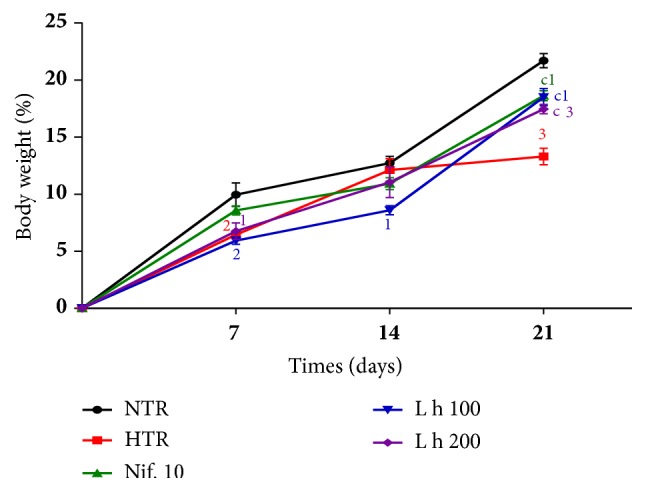
Effects of the aqueous extract of* L. hexandra* on body weight. Each point represents the mean ± SEM (n = 5). NTR: normotensive rats receiving distilled water (10 mL/kg), HTR: hypertensive rats receiving distilled water (10 mL/kg) and alcohol (5 g/kg), L h 100 and L h 200: hypertensive rats receiving the extract of* Leersia hexandra* at doses of 100 and 200 mg/kg, and Nif. 10: hypertensive rats receiving nifedipine at the dose of 10 mg/kg; 1:* p* <0.05, 2:* p* <0.01, and 3:* p* <0.001: significant difference compared to normotensive rats; c:* p* <0.001: significant difference compared to hypertensive rats.

Figure 2 shows the body weight gain of animals during treatment. It appears that hypertensive rats (HTR) receiving distilled water (10 mL/kg) and alcohol (5 g/kg) had a body weight gain significantly lower than that of normotensive rats (NTR), during the first (*p* < 0.01) and third (*p* < 0.001) week of treatment. During the third week of treatment, the body weight of HTR receiving the extract at doses of 100 and 200 mg/kg and HTR receiving nifedipine at the dose of 10 mg/kg was significantly (*p* < 0.001) higher than that of those receiving distilled water and ethanol.

### 3.3. Effects of the Aqueous Extract of* L. hexandra *on Hemodynamic Parameters

As shown in Table 2, systolic blood pressure (SBP), diastolic blood pressure (DBP), and mean blood pressure (MBP) of ethanol-induced HTR receiving distilled water were significantly (*p* < 0.001) increased as compared to normotensive rats (NTR). The administration of* L. hexandra* at the respective doses of 100 and 200 mg/kg as well as nifedipine (10 mg/kg) resulted in a significant reduction (*p* < 0.001) of SBP, DBP, and MBP as compared to HTR. The extract at the dose of 200 mg/kg and nifedipine reduced the values of SBP, DBP, and MBP to values close to those of normotensive rats. The plant extract at both doses (100 and 200 mg/kg) and nifedipine (10 mg/kg) had no effect on the heart rate of hypertensive rats.

### 3.4. Effects of the Aqueous Extract of* L. hexandra *on Lipid Parameters

Table 3 shows the effects of the aqueous extract of* L. hexandra* on the serum level of total cholesterol, triglyceride, HDL-cholesterol, LDL-cholesterol, and the atherogenic index. It shows that total cholesterol, triglyceride, LDL-cholesterol, and the atherogenic index significantly (*p* < 0.001) increased by 49.99%, 57.42%, 118.08%, and 21.00%, respectively, in untreated hypertensive rats as compared to normotensive rats. The significant (*p* < 0.001) decrease in HDL-cholesterol by 52.11% was also observed. The administration of* L. hexandra* extract at the respective doses of 100 and 200 mg/kg or nifedipine (10 mg/kg) resulted in a significant reduction in total cholesterol, respectively, by 17.82% (*p* < 0.05), 20.99% (*p* < 0.01), and 30.81% (*p* < 0.001) as compared to untreated hypertensive rats. The administration of* L. hexandra* extract (100 and 200 mg/kg) or nifedipine (10 mg/kg) also resulted in a significant reduction in triglyceride levels, respectively, by 30.38% (*p* < 0.001), 36.08% (*p* < 0.001), and 32.24% (*p* < 0.001) as compared to untreated hypertensive rats.* L. hexandra* extract (100 and 200 mg/kg) or nifedipine (10 mg/kg) also significantly reduced the levels of LDL-cholesterol, respectively, by 20.56% (*p* < 0.05), 30.01% (*p* < 0.01), and 49.09% (*p* < 0.001) as compared to HTR receiving distilled water. The aqueous extract of* L. hexandra* (100 and 200 mg/kg) or nifedipine (10 mg/kg) also significantly decreased the atherogenic index by 22.92% (*p* < 0.001), 51.55% (*p* < 0.001), and 61.65% (*p* < 0.001) as compared to untreated hypertensive rats. Treatment with* L. hexandra* at the dose of 200 mg/kg or nifedipine (10 mg/kg) resulted in a significant increase in the level of HDL-cholesterol, respectively, by 43.36% (*p* < 0.001) and 80.07% (*p* < 0.001) as compared to untreated hypertensive rats.

### 3.5. Effects of the Aqueous Extract of* Leersia hexandra* on Liver and Kidney Functions

The effects of the aqueous extract of* Leersia hexandra* on liver and kidney functions were evaluated by the assessment of some markers of these functions. As shown in Table 4, daily administration of ethanol for 35 days caused a significant increase (*p* < 0.001) in ALT, AST, and creatinine as compared to normotensive rats. The increase was by 145%, 33.80%, and 344.06%, respectively, for ALT, AST, and creatinine. The plant extract at the dose of 100 mg/kg caused a significant decrease (*p* < 0.001) of ALT, AST, and creatinine by 41.42%, 26.28%, and 90.71%, respectively. At the dose of 200 mg/kg the decrease (*p* < 0.001) of ALT, AST, and creatinine was of 63.80%, 46.42%, and 78.24%, respectively, as compared to untreated hypertensive rats. Nifedipine used in the same condition significantly reduced all these parameters.

### 3.6. Effects of the Aqueous Extract of* Leersia hexandra* on Tissue Protein

**Figure 3:**
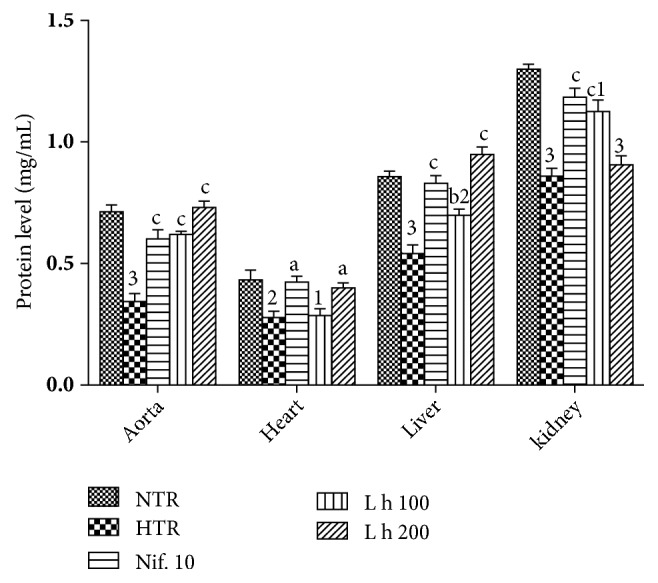
Effects of* Leersia hexandra *aqueous extract on protein levels. Each bar represents mean ± SEM of 5 rats. NTR: normotensive rats receiving distilled water (10 mL/kg), HTR: hypertensive rats receiving distilled water (10 mL/kg) and ethanol (5 g/kg), L h 100 and L h 200: hypertensive rats receiving ethanol and* Leersia hexandra* extract at doses of 100 and 200 mg/kg, and Nif. 10: hypertensive rats receiving nifedipine at the dose of 10 mg/kg; 1:* p* < 0.05, 2:* p* < 0.01, and 3:* p* < 0.001: significant difference from normotensive rats; a:* p* < 0.05, b:* p* < 0.01, and c:* p* < 0.001: significant difference compared to hypertensive rats.

Figure 3 shows the effects of* L. hexandra* on the level of tissue protein. It shows that protein level significantly decreased by 52.11% (*p* < 0.001) in the aorta, by 37.20% (*p* < 0.01) in the heart, by 36.47% (*p* < 0.001) in the liver, and by 34.11% (*p* < 0.001) in the kidneys of untreated hypertensive rats as compared to normotensive rats. Administration of the plant extract (100 mg/kg) resulted in a significant increase in the total protein level by 82.35% (*p* <0.001) in the aorta, by 29.62% (*p* < 0.01) in the liver, and by 31.40% (*p* < 0.001) in the kidneys when compared to untreated hypertensive rats. At the dose of 200 mg/kg, the plant extract significantly increased tissue protein levels by 114.70% (*p* < 0.001) in the aorta, by 42.85% (*p* < 0.05) in the heart, and by 75.92% (*p* < 0.001) in the liver compared with untreated hypertensive rats. Nifedipine used in the same condition as the plant extract significantly increased these parameters in the investigated organs.

### 3.7. Effects of the* Leersia hexandra *Aqueous Extract on Some Markers of Oxidative Stress

**Figure 4:**
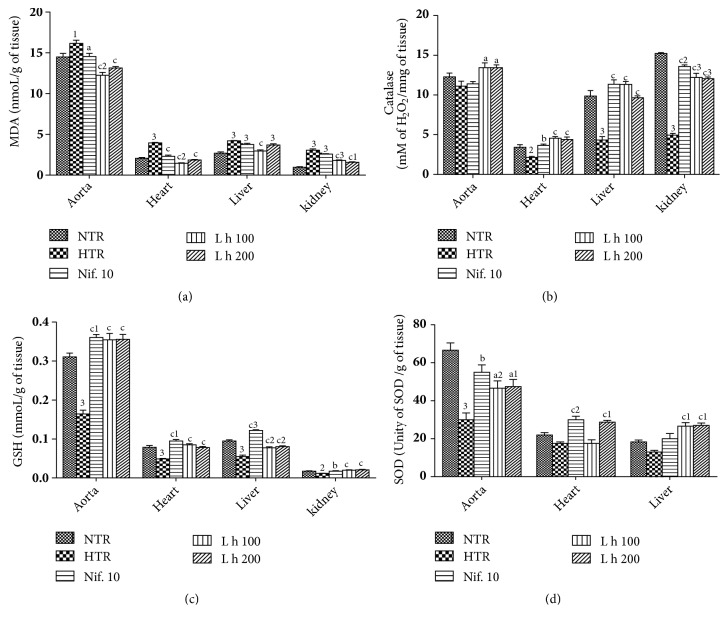
Effects of* Leersia hexandra *aqueous extract on some markers of oxidative stress. Each bar represents mean ± SEM of 5 rats. NTR: normotensive rats receiving distilled water (10 mL/kg), HTR: hypertensive rats receiving distilled water (10 mL/kg) and ethanol (5 g/kg), L h 100 and L h 200: hypertensive rats receiving ethanol and* Leersia hexandra* extract at doses of 100 and 200 mg/kg, and Nif. 10: hypertensive rats receiving nifedipine at the dose of 10 mg/kg; 1:* p* < 0.05, 2:* p* < 0.01, and 3:* p* < 0.001: significant difference from normotensive rats; a:* p* < 0.05, b:* p* < 0.01, and c:* p* < 0.001: significant difference compared to hypertensive rats.

The effects of* Leersia hexandra* aqueous extract on some markers of oxidative stress are shown in Figure 4. Treatment with alcohol induced a significant increase (*p* < 0.001) in the aorta, heart, liver, and kidneys MDA concentration, respectively, by 11.45%, 93.20%, 56.35%, and 192.74% as compared to normotensive rats (Figure 3(a)). The plant extract significantly reduced (*p* < 0.001) MDA concentration in the aorta (24.21% and 18.63%), in the heart (63.90% and 53.38%), and in the kidneys (39.59% and 47.31%), respectively, at doses of 100 and 200 mg/kg as compared to untreated hypertensive rats. Treatment with alcohol induced a significant decrease in catalase activity by 37.42% (*p* < 0.01) in the heart, by 56.03% (*p* < 0.001) in the liver, and by 67.41% (*p* < 0.001) in the kidneys as compared to normotensive rats (Figure 3(b)). The plant extract significantly increased catalase activity by 21.06% (*p* < 0.05) in the aorta, by 112.61% (*p* < 0.001) in the heart, by 161.43% (*p* < 0.001) in the liver, and by 146.16% (*p* < 0.001) in to the kidneys at the dose of 100 mg/kg as compared hypertensive rats. At the dose of 200 mg/kg, an increase was observed in catalase activity by 21.06% (*p* < 0.05) in the aorta, by 105.14% (*p* < 0.001) in the heart, by 122.35% (*p* < 0.001) in the liver, and by 143.14% (*p* < 0.001) in the kidneys. Alcohol induced a significant increase (*p*<0.001) of GSH concentration in the aorta, heart, liver, and kidneys, respectively, by 48.38%, 42.85%, 44.44%, and 35.29% as compared to normotensive rats (Figure 3(c)). The extract induced a significant decrease (*p* < 0.001) in GSH concentration in the aorta (118.75% and 118.75%), in the heart (80.00% and 60.00%), in the liver (33.33% and 33.33%), and in the kidneys (100.00% and 100.00%), respectively, at doses of 100 and 200 mg/kg as compared to untreated hypertensive rats. Ethanol induced significant decrease (*p* < 0.001) in SOD activity in the aorta (55.55%) as compared to normotensive rats (Figure 3(d)). The plant extract induced a significant increase in SOD activity by 55.57% (*p* < 0.05) in the aorta and by 105.15% (*p* < 0.001) in the liver at the dose of 100 mg/kg as compared to hypertensive rats. At the dose of 200 mg/kg, an increase was observed in SOD activity by 58.33% (*p* < 0.05) in the aorta, by 64.29% (*p* < 0.001) in the heart, and by 107.69% (*p* < 0.001) in the liver.

### 3.8. Effects of the Aqueous Extract of* Leersia hexandra* on the Histoarchitecture of the Aorta

**Figure 5:**
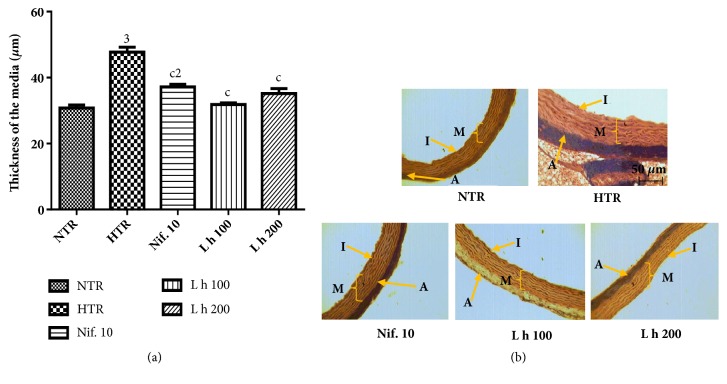
Effects of* Leersia hexandra *aqueous extract on histomorphometry (a) and histology (b) of the aorta (hematoxylin and eosin, x100). Each bar represents mean ± SEM of 5 rats. NTR: normotensive rats receiving distilled water (10 mL/kg), HTR: hypertensive rats receiving distilled water (10 mL/kg) and ethanol (5 g/kg), L h 100 and L h 200: hypertensive rats receiving ethanol and extract of* Leersia hexandra* at doses of 100 and 200 mg/kg, and Nif. 10: hypertensive rats receiving nifedipine at the dose of 10 mg/kg;** A**: adventitia,** I**: intima, and** M**: media; 2:* p* < 0.01 and 3:* p* < 0.001: significant difference compared to normotensive rats; c:* p* < 0.001: significant difference compared to hypertensive rats.

Figure 5 shows the effects of* L. hexandra* on histomorphometry (a) and histology (b) of the aorta. Treatment with alcohol induced a significant increase of thickness of the media by 54.95% (*p* < 0.001) as compared to normotensive rats. The plant extract (100 and 200 mg/kg) reduced significantly (*p* < 0.001) the thickness of that tunica, respectively, by 33.30% and 26.24% as compared to hypertensive rats.

## 4. Discussion

The antihypertensive effects of the aqueous extract of* Leersia hexandra* were evaluated in ethanol-induced hypertensive rats. Ethanol has been shown to promote hemodynamic disturbances that cause high blood pressure [15]. In the present study, administration of ethanol 40° at the dose of 5 g/kg/day for 8 weeks resulted in a significant increase in systolic, diastolic, and mean blood pressure as well as heart rate. Several mechanisms are responsible for installing this HTA [16]. Chronic consumption of alcohol has been shown to induce cardiovascular dysfunction and therefore is the major contributory factor of hypertension [15]. Abidemi et al. [17] reported that ethanol has complex direct vascular effects, including basal constriction and prominent elastic lamellae fragmentation, which affect vessels' elasticity. Vascular dysfunction due to ethanol may induce changes in the relaxant capacity and decrease the endothelial release of nitric oxide. Administration of* Leersia hexandra* extract or nifedipine significantly reduced blood pressure in hypertensive rats. These results suggest that the aqueous extract of* L. hexandra* may have an antihypertensive effect that could be due to an action on peripheral resistance.

In untreated hypertensive rats, the increase in blood pressure was accompanied by a reduction of weight gain and a significant decrease in tissue protein levels in the aorta, heart, liver, and kidneys. This could be due to the deleterious effects caused by alcohol in the cells [18]. According to those authors, alcohol prevents the absorption of nutrients by destroying the cells that cover the stomach and intestine. Alcohol also inhibits the oxidation of nutrients by reducing the secretion of digestive enzymes by the pancreas [19]. Like nifedipine, the aqueous extract of* Leersia hexandra* significantly increased the level of protein in these different organs and also stimulated weight gain at the end of treatment as compared to hypertensive rats receiving distilled water. These results suggest that* L. hexandra* would be able to protect the structural integrity of cells against the attacks of toxic molecules derived from the metabolism of ethanol.

Dyslipidemia is a significant and independent risk factor of cardiovascular disease. Ethanol treated rats showed a significant decrease in HDL-cholesterol and increase in total cholesterol, triglycerides, and LDL-cholesterol levels as well as the atherogenic index. These findings are in agreement with results obtained by some authors who reported that hypercholesterolemia and dyslipidemia are associated with the pathogenesis of hypertension induced by chronic ethanol and sucrose intake or L-NAME [20, 21]. According to Stevens et al. [22], dyslipidemia is a major risk factor for atherosclerosis that promotes increased vascular resistance and contributes to increased blood pressure. This is justified in our study by the high levels of the atherogenic index. Like nifedipine, the aqueous extract of* L. hexandra* significantly reduced serum triglyceride, total cholesterol, LDL-cholesterol, and atherogenic index levels in addition to an increase in HDL-cholesterol levels. These results suggest that* L. hexandra* extract contains substances that could correct dyslipidemia in alcohol-induced hypertension.

Chronic diseases like hypertension are always accompanied by oxidative stress responsible for their complication. High consumption of alcohol is associated with oxidative stress and increased level of free radicals, which have been reported to play an important role in the pathogenesis of hypertension [17]. Analysis of oxidative stress parameters showed that malondialdehyde levels increased significantly in the aorta, heart, liver, and kidneys of hypertensive rats receiving distilled water and ethanol. The high level of MDA, the most important marker of lipid peroxidation in HTR, is a sign of cell membrane alteration, thus promoting the leaking of enzymes into the bloodstream [23]. The significant decrease in reduced glutathione levels in the investigated organs in hypertensive rats corroborates the findings of Aboubakar et al. [15] who showed that ethanol depending on the dose increased the level of MDA and reduced that of reduced glutathione. The metabolism of alcohol is closely related to the production of reactive oxygen species leading to oxidative stress [16]. Reduced glutathione is an endogenous, nonenzymatic antioxidant in its oxidized form; it acts by directly trapping reactive oxygen species [24]. The decrease in GSH observed in the present study is obviously related to ethanol-induced oxidative stress, which is characterized by the production of acetaldehyde (toxic metabolite) and other reactive molecules in the cell [25]. The treatment of hypertensive rats with the plant extract significantly increased the concentration of GSH and reduced that of MDA in tissue, suggesting that* L. hexandra* extract may act by directly trapping free radicals and/or reducing their production, thus limiting the use of GSH. These results are in conformity with some works [20, 26] which showed that the antioxidant activity of some plant extracts is related to the increase in GSH level. Our results also showed that ethanol significantly reduced the activity of superoxide dismutase (SOD) as well that of as catalase. SOD and catalase are important enzymes, which protect cells against free radical injury mediated by O_2_^–^ and H_2_O_2_ [15]. The decrease in their activity may be due to the excessive ROS production [15]. The aqueous extract of* L. hexandra* as well as nifedipine improved the antioxidant status in hypertensive rats. These results suggest that* L. hexandra* has an antioxidant effect that protects the tissues from the deleterious effects of free radicals resulting from the metabolism of ethanol. This was confirmed by the investigation of liver and kidneys functions. In the present study, alcohol-induced hypertension was characterized by the increase in ALT and AST activity as well as creatinine level. This may be the result of membrane alteration due to lipid peroxidation. Similar results were obtained by Aboubakar et al. [15] and Bilanda et al. [26], justifying the side effect of alcohol and oxidative stress on liver and kidney cells.

## 5. Conclusion

*Leersia hexandra* aqueous extract exhibited an antihypertensive action in ethanol-induced hypertension. This action may be related to the antioxidative and lipid lowering effect. This was associated with improvement of liver and kidneys functions. These properties justify the empirical use of* L. hexandra* in the treatment of hypertension. More study needs to be done in order to identify the compounds isolated in the extract.

## Figures and Tables

**Table 1:** Main signals exhibited in the HPLC-DAD-MS spectra of compounds detected in *L. hexandra *and proposed attribution.

**N**°	**Tr (min)**	**M+H**	**Name of compound**
**01**	9.50	232.94	NI
**02**	3.80	213.16	NI
**03**	3.70	213.16	NI
**04**	1.30	181.08	Glucose

NI: not identified; Tr: retention time.

**Table 2:** Effects of the aqueous extract of *L. hexandra *on hemodynamic parameters.

**Parameters**	**NTR**	**HTR**	**Nif. 10**	**L h 100**	**L h 200**
**SBP (mmHg)**	112.50 ± 0.86	175.02 ± 2.64^3^	118.50 ± 2.25 ^c^	100.20 ± 1.83 ^c1^	117.20 ± 1.13 ^c^
**DBP (mmHg)**	101.53 ± 1.54	160.52 ± 2.67^3^	105.40 ± 1.81 ^c^	91.08 ± 3.73 ^c1^	104.40 ± 1.97 ^c^
**MBP (mmHg)**	105.20 ± 1.50	165.35 ± 2.64^3^	109.80 ± 0.85 ^c^	94.13 ± 2.51 ^c^	108.70 ± 1.28 ^c^
**HR (BPM)**	320.10 ± 1.60	346.20 ± 2.33^3^	347.83 ± 1.15^3^	347.70 ± 2.20^3^	345.60 ± 1.18^3^

Each value represents the mean ± SEM (n = 5). BPM: beat per minute, SBP: systolic blood pressure, DBP: diastolic blood pressure, MBP: mean blood pressure, HR: heart rate, and NTR: normotensive rats receiving distilled water (10 mL/kg), HRT: hypertensive rats receiving distilled water (10 mL/kg) and alcohol (5 g/kg), L h 100 and L h 200: hypertensive rats receiving ethanol and *Leersia hexandra* extract at the doses of 100 and 200 mg/kg, respectively, and Nif. 10: hypertensive rats receiving alcohol and nifedipine at the dose of 10 mg/kg; 1: *p* <0.05 and 3: *p* <0.001: significant difference from normotensive rats; c: *p* <0.001: significant difference from hypertensive rats.

**Table 3:** Effects of aqueous extract of *L. hexandra* on lipid parameters.

**Parameters**	**NTR**	**HTR**	**Nif. 10**	**L h 100**	**L h 200**
**TC (mg/dL)**	149.36 ± 8.05	224.02 ± 5.45^3^	154.98 ± 11.20^c^	184.09 ± 7.59^a1^	176.98 ± 6.62^b^
**TG (mg/dL)**	63.74 ± 0.63	100.34 ± 0.38^3^	67.99 ± 0.20^c3^	69.86 ± 1.83^c3^	64.13 ± 0.23^c^
**LDL-Chol (mg/dL)**	76.04 ± 9.51	175.12 ± 5.74^3^	89.14 ± 9.42^c^	139.11 ± 7.51^a3^	122.56 ± 7.02^b2^
**HDL-Chol (mg/dL)**	60.58 ± 2.41	29.01 ± 0.50^3^	52.24 ± 1.28^c2^	31.01 ± 0.68^3^	41.59 ± 0.65^c3^
**Atherogenic index**	2.49 ± 0.21	7.72 ± 0.14^3^	2.96 ± 0.15^c^	5.95 ± 0.30^c3^	3.74 ± 0.12^c2^

Each value represents the mean ± SEM (n = 5). TC: total cholesterol, LDL-Chol: LDL-cholesterol, HDL-Chol: HDL-cholesterol, TG: triglycerides, and NTR: normotensive rats receiving distilled water (10 mL/kg), HTR: hypertensive rats receiving distilled water (10 mL/kg) and ethanol (5 g/kg), L h 100 and L h 200: hypertensive rats receiving ethanol and *Leersia hexandra* extract at doses of 100 and 200 mg/kg, and Nif. 10: hypertensive rats receiving ethanol and nifedipine at the dose of 10 mg/kg; 1: *p* <0.05, 2: *p* <0.01, and 3: *p* <0.001: significant difference from normotensive rats; a: *p* <0.05, b: *p* <0.01, and c: *p* <0.001: significant difference compared to hypertensive rats.

**Table 4:** Effects of the aqueous extract of *Leersia hexandra* on liver and kidney functions.

**Parameters**	**NTR**	**HTR**	**Nif. 10**	**L h 100**	**L h 200**
**ALT (U/L)**	22.84 ± 1.29	30.56 ± 2.24^2^	15.71 ± 0.95^c2^	17.90 ± 0.62^c^	11.06 ± 0.49^c3^
**AST (U/L)**	133.47±2.13	327.01±1.45^3^	175.18 ±7.28^c2^	241.04 ±13.73^c3^	239.78±0.59^c3^
**Creatinine (mg/dL)**	1.77 ± 0.15	7.86 ± 0.49^3^	2.57 ± 0.40^c^	0.73 ± 0.28^c^	1.71 ± 0.20^c^

Each value represents the mean ± SEM (n = 5). NTR: normotensive rats receiving distilled water (10 mL/kg), HTR: hypertensive rats receiving distilled water (10 mL/kg) and ethanol (5 g/kg), L h 100 and L h 200: hypertensive rats receiving ethanol and *Leersia hexandra* extract at doses of 100 and 200 mg/kg, and Nif. 10: hypertensive rats receiving nifedipine at the dose of 10 mg/kg; 2: *p* <0.01 and 3: *p* <0.001: significant difference compared to the normal control; c: *p* <0.001: significant difference compared to hypertensive rats.

## Data Availability

The data used to support the findings of this study are available from the corresponding author upon request.
